# The experience of transition to adult services of young people with neurodevelopmental disorders and mental health problems

**DOI:** 10.1192/j.eurpsy.2025.110

**Published:** 2025-08-26

**Authors:** J. Santambrogio, M. Marchetti, Alessandra Beretta, Erika Morandi, Francesca Formaggio, Ilaria Ruberto, Milena Danese, Giuseppina Redaelli, Massimo Clerici, Sergio Terrevazzi, Antonio Amatulli, Armando D’Agostino

**Affiliations:** 1 Department of Mental Health and Addiction, Disability Unit, ASST Brianza, Limbiate; 2 Department of Mental Health and Addiction, ASST Valle Olona, Busto Arsizio; 3 Department of Health Sciences, University of Milan, Milano, Italy

## Abstract

**Background:**

Transitional care refers to the coordination and continuity of care between different healthcare locations or levels of care within the same facility, regardless of the patient’s age. Transition planning and management is therefore a key element in the organization and delivery of health services. Unfortunately, for many young people with mental health problems, transition is poorly planned, lacks coordination, and results in discontinuity of care. This is particularly true for intellectual disability, Autism Spectrum Disorder, and ADHD: as neurodevelopmental conditions, care typically begins at a young age with attendance at Child and Adolescent Mental Health Services (CAMHS), and the transition to Community Mental Health Services (CMHS) encounters numerous challenges.

**Methods:**

A narrative review of the literature was conducted on the topic of transition from inception to January 26th, 2025. The following search string was used through PubMed, Web of Science, Scopus, and PsychInfo: (**“**Transitional Care**”** OR **“**Transition to Adult Care**”**) AND (**“**Neurodevelopmental Disorders**”** OR **“**Intellectual Disability**”** OR **“**Autistic Disorder**”** OR **“**Autism Spectrum Disorder**”**) AND (**“**Child and Adolescent Mental Health Services**”** OR **“**CAMHS**”** OR **“**Community Mental Health Centers**”**).

**Results:**

A total of 202 references were identified and screened considering titles and abstracts. After excluding papers not relevant to the topic and those that were unretrievable, 31 papers were included in the review. A significant majority of the included papers were qualitative studies based on focus groups and interviews conducted with family members and caregivers.

**Discussion and conclusions:**

Findings reveal a lack of clarity and consistency regarding service availability for the conditions studied, possibly due to different eligibility criteria between child and adult mental health services, with variable service provision for young people with neurodevelopmental conditions (specifically, ASD, ADHD and ID). While high workloads and staff shortages were perceived to influence service thresholds and eligibility criteria, the lack of perceived severity by the CMHS often led to loss of follow-up.

**Disclosure of Interest:**

None Declared

